# Validation of the Arabic version of the Brief Emotional Intelligence Scale

**DOI:** 10.3389/fpsyg.2026.1676270

**Published:** 2026-02-10

**Authors:** Souheil Hallit, Sahar Obeid, Gaelle Kanj, Diana Malaeb, Kamel Jebreen, Vanessa Azzi, Rabih Hallit, Feten Fekih-Romdhane

**Affiliations:** 1 School of Medicine and Medical Sciences, Holy Spirit University of Kaslik, Jounieh, Lebanon; 2 Applied Science Research Center, Applied Science Private University, Amman, Jordan; 3 Department of Psychology and Education, School of Arts and Sciences, Lebanese American University, Jbeil, Lebanon; 4 College of Pharmacy, Gulf Medical University, Ajman, United Arab Emirates; 5 Department of Mathematics, Palestine Technical University – Kadoorie, Hebron, Palestine; 6 Department of Infectious Disease, Bellevue Medical Center, Mansourieh, Lebanon; 7 Department of Infectious Disease, Notre Dame des Secours, University Hospital Center, Byblos, Lebanon; 8 The Tunisian Center of Early Intervention in Psychosis, Department of Psychiatry “Ibn Omrane”, Razi Hospital, Manouba, Tunisia; 9 Faculty of Medicine of Tunis, Tunis El Manar University, Tunis, Tunisia

**Keywords:** Arabic, brief measure, confirmatory factor analysis, emotional intelligence, psychometric properties

## Abstract

**Background:**

Emotional Intelligence (EI) has been a widespread topic of scientific research and discussion among individuals and institutions over the last years, giving room for debate concerning its measurement and applications. One of the shortest, simplest, most economical and increasingly used self-report measures is the Brief Emotional Intelligence Scale (BEIS-10), whose validity and reliability have been demonstrated across diverse samples and contexts. The present study examined the psychometric properties of an Arabic translation of the BEIS-10 in a native Arabic-speaking population from Lebanon.

**Methods:**

This cross-sectional study enrolled 449 non-clinical adults (median age = 21 years [min = 18; max = 70]; 70.6% females; median household crowding index = 1 [min = 0.14; max = 8]). The forward-backward translation approach was adopted to develop an Arabic version of the BEIS-10. We used data from the entire sample to perform a Confirmatory Factor Analysis (CFA) using RStudio, “lavaan” and “SemTools” programs. We used the Weighted Least Squares with Mean and Variance (WLSMV) estimation method, which is more appropriate for ordinal data.

**Results:**

CFA indicated that although both the one-factor and five-factor models of the BEIS-10 demonstrated acceptable global fit indices, inspection of the five-factor solution revealed extremely high inter-factor correlations, several approaching unity, indicating poor discriminant validity among the proposed dimensions. Consequently, the unidimensional model of the Arabic BEIS-10 was retained, showing excellent internal consistency as evidenced by McDonald’s *ω* and Cronbach’s *α* values of 0.94. The Arabic BEIS-10 showed invariance across gender at the scalar, metric, and configural levels, with no difference between genders in terms of EI. Furthermore, higher EI scores significantly correlated with higher positive and negative affect, thus attesting to the concurrent validity of the Arabic BEIS-10.

**Conclusion:**

Findings suggest that the Arabic BEIS-10 is a valid and reliable self-report measure for the assessment of EI in Arab settings. Considering its simple and brief administration, this version of the scale might be of better convenience in Arab settings beset by shortage of resources and financial turmoil. It also has practical implications as it enables research on EI in diverse cultural contexts, and enhances educational and workplace programs by tailoring emotional intelligence training to local needs.

## Introduction

Emotional Intelligence (EI) emerged in the 1990s as a significant construct in the domain of psychology that refers to the “ability to monitor one’s own and others’ feelings and emotions, to discriminate among them and to use this information to guide one’s thinking and actions” (Salovey and Mayer, 1990, p. 189). Possessing high levels of EI translates into owning emotional skills and capacities that permit individuals to accurately perceive and effectively regulate emotions (e.g., anger, sadness) in themselves as well as in others (Salovey and Mayer, 1990). Subsequently, highly emotionally intelligent individuals are likely to achieve a set of adaptive emotional states or other advantageous outcomes, including enhanced creative thinking, and motivation. There is a general agreement that the construct of EI can be divided into two models, of which the trait and ability models are deemed predominant (Mayer et al., 2008; Li and Xu, 2019). EI’s trait models refer to an understanding of intelligence that is broader, combining dispositional behavior and social skills (Extremera and Rey, 2016), while EI’s ability models conceptualize EI as the capacity to perceive, access, understand and manage emotions (Extremera and Rey, 2016). A third mixed model designates a range of perspectives on the extent to which EI is perceived either as an ability that can be developed or as affect-related personality traits (Mayer, 1999; Cherniss, 2010; Brackett et al., 2011). This distinction is crucial for model specification, as BEIS-10 aligns more closely with trait EI, which supports its use in self-report measurement models. Given that trait EI reflects stable emotional dispositions and competencies expressed in typical behavior, self-report tools like the BEIS-10 are theoretically appropriate for its measurement. Therefore, our decision to validate the scale using Confirmatory Factor Analysis (CFA) is aligned with the conceptualization of trait EI as captured by the BEIS-10.

A number of meta-analyses have highlighted that individuals with higher EI levels tend to have a better health (Martins et al., 2010) and subjective wellbeing (Sánchez-Álvarez et al., 2016). In particular, EI is consistently found to predict better performance at the workplace and in the academic setting (MacCann et al., 2020; Joseph et al., 2015; O'Boyle et al., 2011), higher job satisfaction, higher organizational commitment, and lower turnover intentions (Miao et al., 2017), higher achievement of optimal sports’ performance in competitive sports (Kopp and Jekauc, 2018), higher levels of social support (Sarrionandia and Mikolajczak, 2020), better decision making (El Othman et al., 2020), and greater romantic relationship satisfaction (Malouff et al., 2010). On the other hand, there is sufficient evidence that EI is inversely associated with a range of psychopathology indicators, including occupational stress and burnout (Vashisht et al., 2018; Lahoud et al., 2019), disordered eating behaviors (Zhang et al., 2022), alcohol misuse and alcohol-use-related problems (Peterson et al., 2011), aggressive behaviors (Vega et al., 2022), sleep quality, physical activity, substance use (Sarrionandia and Mikolajczak, 2020), and maladaptive personality traits (Miao et al., 2019). In light of these observations, researchers recommended that organizations incorporate EI in employees’ recruitment and training programs (Miao et al., 2017). This emphasizes the major importance of valid assessment tools to evaluate and monitor EI in different settings, thereby informing evidence-based policies and practices.

Despite this generally adaptive profile, emerging evidence suggests that the relationship between EI and affective experiences may be more complex than traditionally assumed (Mikolajczak et al., 2008). In particular, trait-based conceptualization of EI emphasize heightened emotional awareness, sensitivity, and attentional focus toward emotional states rather than emotional valence alone (Mikolajczak et al., 2008). Individuals with higher trait EI may therefore experience and report emotions, both positive and negative, with greater intensity and clarity (Barrett et al., 2001; Thompson et al., 2011). From this perspective, elevated negative affect among individuals with higher EI does not necessarily reflect poorer emotional functioning, but rather increased emotional insight, openness, and accuracy in identifying internal states (Palmieri et al., 2009). Such findings challenge a unidirectional interpretation of EI as solely protective against negative affect and suggest that emotionally intelligent individuals may be more attuned to their emotional experiences across the full affective spectrum (Zeidner et al., 2012a).

Various measuring tools have been developed over the years, particularly characterizing early research on the EI construct, such as the Mayer-Salovey-Caruso Emotional Intelligence Test (MSCEIT; original and updated versions, e.g., MSCEIT v2.0) (Skaar, 2007), the Self-report Emotional Intelligence Test (SREIT) (Schutte et al., 1998), the Bar-On Emotional Quotient Inventory (EQ-i and its revised versions, including EQ-I 2.0) (Bar-On, 1997), and the Emotional and Social Competence Inventory (ESCI) (Boyatzis and Goleman, 2007). Most of these measures are based on larger definitions of EI that involve adaptive emotional functioning and outcomes (Schutte et al., 2009). One of the most popular and widely employed measures is the Assessing Emotions Scale (AES) (Salovey and Mayer, 1990). However, these tools are often lengthy, complex to administer, or require specific training and resources, which can limit their use. In contrast, the BEIS-10 offers a shorter, more accessible alternative that focuses on behavioral aspects of EI, making it suitable for research settings where brevity and ease of use are essential.

Considered a trait measure of EI, the AES is based on the original model of EI of Salovey and Mayer (1990), which proposes EI reflects appraisal, expression, and regulation of emotion in the self and others, as well as utilization of emotion in solving problems. The scale was initially designed to assess EI as a unidimensional construct (Schutte et al., 1998), using total scores. Previous psychometric studies focusing on the factorial validity of the AES have confirmed either a single-factor solution (Brackett and Mayer, 2003; Ciarrochi et al., 2001), or a higher order factor with related sub-factors (Gignac et al., 2005), a three-factor (Austin et al., 2004; Kun et al., 2010) or a four-factor solution (Davies et al., 2010; Saklofske et al., 2003; Satuf et al., 2020; Hallit et al., 2023; Omar et al., 2014). However, overall, the vast majority of validation studies focused on exploring or confirming the factor structure of the 33-item AES, and showed an instability regarding the organization of its factors. Consequently, Davies et al. (2010) proposed a shortened version of the AES, composed of only 10 items and labeled the Brief Emotional Intelligence Scale (BEIS-10). The BEIS-10 was obtained after discarding items that were considered insignificant and theoretically redundant, which may partly address the above-mentioned issues related to the inconsistency of the AES’s factor structure. Davies et al. (2010) applied a Confirmatory Factor Analysis (CFA) to both a unidimensional and a theoretically-based 5-factor models in a student-athlete population. Findings revealed the goodness of fit of the 5-factor solution, with each factor having two items (i.e., Appraisal of own emotions, Appraisal of others’ emotions, Regulation of own emotions, Regulation of others’ emotions, and Utilization of emotion) (Davies et al., 2010). However, the factors were highly correlated, thereby supporting the use of a total EI score. Davies et al. (2010) recognized an urgent need to test the stability of this factor structure in other population groups, given the well-established methodological limitations related to performing a CFA on measures with two items per factor.

Importantly, because trait EI is commonly assessed using self-report instruments, observed associations with affective constructs may partly reflect shared method variance and conceptual overlap (Davies et al., 1998; Podsakoff et al., 2003), particularly with dimensions such as emotional awareness, affect intensity, and mood monitoring (Davies et al., 1998). Unlike ability-based EI measures, self-report trait EI captures individuals’ perceptions of their emotional competencies, which may be influenced by dispositional tendencies toward emotional introspection and expressiveness (Petrides and Furnham, 2001). As a result, individuals scoring high on trait EI may report elevated levels of both positive and negative affect, not due to ineffective emotion regulation, but rather because of heightened awareness and acknowledgment of their emotional experiences (Mikolajczak et al., 2008). This distinction is critical when interpreting correlations between EI and affective outcomes and underscores the need for cautions and theory-driven interpretation of such findings (Salovey and Grewal, 2005).

Beyond its demonstrated psychometric qualities, the BEIS-10 offers many advantages over the full-length version because of its briefness, including low-cost, reduced administration time (it only takes 1–2 min to complete), and less respondents’ burden thus avoiding non-response. Therefore, the BEIS-10 is appropriate for use in clinical and research settings with time constraints or limited resources, such as those in the Arab developing countries. The brief form is also beneficial in large-scale surveys with numerous measures to complete or multiple time points. Additionally, shorter scales with a small number of items are more convenient and tolerable for respondents in the increasingly used online surveys, who are less willing to bear the same degree of response burden than those in face-to-face surveys (Granello and Wheaton, 2004). Moreover, the shortness of instruments is emphasized as elementary in research seeking to maintain an increased degree of ecological validity in settings such as athletic competition and academic examinations (Lane, 2007). In light of these advantages, the BEIS-10 has attracted international attention and increased use in research since its publication. Subsequent linguistic validations have emerged examining the psychometric properties of the scale in different countries and contexts, including Italian (Durosini et al., 2021), Iranian (Hadadian-Chaghaei et al., 2021), Canadian (Balakrishnan and Saklofske, 2015), Spanish (Martín and Guzmán, 2012). However, to the best of the researchers’ knowledge, no studies have explored the psychometric properties of the BEIS-10 in the Arabic language and context to date. Moreover, since prior validation studies of the AES showed inconsistent factor structures across cultures and contexts, a short-form like BEIS-10 presents a more stable and replicable alternative, warranting investigation in culturally distinct populations such as Arabic-speaking groups.

There is sufficient evidence that EI is culturally-dependent, varying widely between collectivist and individualist societies (Fukuda et al., 2011; Fukuda et al., 2012; Li et al., 2012; Walter et al., 2021). Consequently, specific cultural dimensions are antecedents of EI. Individuals from countries scoring high on collectivism seem to be more emotionally intelligent (Gunkel et al., 2014). A collectivistic society expects cohesion with peers and, therefore, individual emotions are controlled; even though own emotions are recognized, they might be suppressed for the benefit of the collective (Gunkel et al., 2014). Since emotions are not shown openly, it is also difficult to observe and recognize emotions of others. Conversely, in individualist cultures, EI emphasizes self-awareness, self-expression, and personal achievement (Vishkin et al., 2023).

Within collectivistic cultural contexts such as those prevalent in many Arab societies, emotional intelligence may manifest through heightened emotional sensitivity and internal regulation rather than overt emotional expression (Matsumoto et al., 2008). While individuals may be adept at recognizing both positive and negative emotions, cultural norms often encourage the modulation or internalization of negative affect to preserve social harmony (Matsumoto et al., 2008). As a result, emotionally intelligent individuals in such contexts may report higher levels of negative affect in self-report measures, reflecting emotional awareness rather than maladaptive functioning (Lane and Smith, 2021). These cultural dynamics may partially explain non-traditional associations between EI and affective dimensions observed in Arabic-speaking populations and highlight the importance of interpreting EI-affect relationships within their sociocultural framework (Triandis, 2001).

Furthermore, studies have shown that cultural orientations, such as individualism and collectivism, are associated with varying levels of EI, mental health, and life satisfaction, with collectivist orientations often correlating with higher EI and better mental health outcomes (Bhullar et al., 2012). In this regard, EI should only be interpreted within a cultural context, taking into account one might be determined as emotionally intelligent in one cultural context and not another (Sibia et al., 2003; Brackett and Geher, 2006). However, there is a lack of empirical evidence on EI in Arab countries, which may represent a major impediment to international research progress in this field. Although an Arabic validated version of the full-length 33-item AES exists (Hallit et al., 2023), we believe that making a shorter and more economic form of the scale available in the Arabic language, while preserving its psychometric properties, will substantially lessen the effort and time put into its completion, which may, in turn, increase data quality and response rates (Franke et al., 2013). This could be of great utility and convenience to both Arab researchers and respondents, and may potentially foster research in this field in the Arab region. On these bases, our study sought to examine the psychometric properties of an Arabic translation of the BEIS-10 in a native Arabic-speaking population from Lebanon [i.e., (1) the factorial validity to confirm its underlying structure; (2) Measurement invariance across gender to ensure the scale operates equivalently for both males and females; (3) Internal reliability to assess the consistency of the scale’s components; and (4) Convergent and discriminant validity to examine how the scale positively correlates with related constructs while showing weak correlations with unrelated constructs, thereby supporting its overall construct validity]. The selection of these psychometric evaluations is grounded in classical test theory and recent recommendations for validating brief psychological scales. Factorial validity is essential to confirm whether the theoretical model replicates in a new cultural context; gender invariance ensures fair use of the scale across sexes; reliability tests internal consistency of the latent construct and convergent and discriminant validity allow for construct validation through meaningful correlations with established affective constructs. We expected that the Arabic BEIS-10 will replicate the originally proposed factor structure, and demonstrate measurement invariance across gender groups, good internal consistency, and concurrent, discriminant validity.

## Methods

### 2.1 Study design and participants

This cross-sectional study was carried out between December 2020 and January 2021; 449 participants filled out an online questionnaire using a Google Forms. The survey was anonymous, and participants completed the survey voluntarily and without remuneration. A snowball technique was implemented across Lebanese governorates to collect the aforementioned sample. We chose to use the snowball sampling primarily due to logistical constraints, particularly the limited availability of funds. While the target population of adults over 18 years old residing in Lebanon may not be considered “hard to reach”, snowball sampling was still an appropriate method for our study for several reasons: (1) access to diverse participants (specific regions, socioeconomic backgrounds, etc.), which allows for better access to a range of individuals who might not otherwise be easily reached through conventional methods, and (2) given the limited funding, traditional probability sampling techniques would have been costly and time-consuming. The inclusion criteria consisted of adults over 18 years old, residing in Lebanon, with internet access, and the ability to read and understand Arabic. Exclusion criteria were individuals who declined to take part in this study. Before filling out the questionnaire, participants were provided with an overview of the study objective and were assured of their anonymity in response. After providing digital informed consent, participants were asked to complete the instruments described above.

Four hundred forty-nine participants completed the survey, with a mean age of 24.34 ± 8.22 years, 70.6% females and 78.8% with a university education level. This demographic distribution indicates an overrepresentation of women and highly educated individuals in the sample. These characteristics should be kept in mind when interpreting the findings, as they may not fully reflect the broader Lebanese adult population. The remaining details can be found in Table 1.

**Table 1:** Sociodemographic characteristics of the participants (*n* = 449).

Variables	Mean ± SD or *N* (%)
Age (years)	24.34 ± 8.22
Household crowding index (person/room)	1.16 ± 0.66
Gender	
Male	132 (29.4%)
Female	317 (70.6%)
Education level	
Secondary or less	95 (21.2%)
University	354 (78.8%)
Marital status	
Single	364 (81.1%)
Married	85 (18.9%)

### 2.2 Minimum sample size

A previous study suggested that the minimum sample size to conduct a CFA ranges from 3 to 20 times the number of the scale’s variables (Mundfrom et al., 2005). Therefore, we assumed a minimum sample of 200 participants needed to have enough statistical power based on a ratio of 20 participants per one item of the scale, which was exceeded in our sample.

### 2.3 Human ethics and consent to participate

This study protocol was performed in accordance with the relevant guidelines and regulations. The *Psychiatric Hospital of the Cross Ethics committee* approved it (HPC-044–2020). All methods were carried out in accordance with relevant guidelines and regulations/Declaration of Helsinki. Submitting the form online was equivalent to obtaining a written informed consent from each participant.

### 2.4 Data collection and instruments used

The Arabic language was employed in the questionnaire. A sociodemographic section was included in the questionnaire in addition to a scale-based category as detailed below:

#### 2.4.1 Sociodemographic data

This category of the questionnaire collected general sociodemographic data about individual respondents, including age, gender, marital status, educational level and household crowding index reflecting the socioeconomic status; the latter was calculated by dividing the number of persons by that of the rooms in the house except the bathrooms and kitchen (Melki et al., 2004). The following scales were used in the questionnaire.

#### 2.4.2 The Brief Emotional Intelligence Scale (BEIS-10)

This scale assesses trait EI through 10 items divided into five factors: (1) “appraisal of own emotions”, which evaluates individuals’ ability to identify emotions factors leading to changing in emotions in themselves; (2) “appraisal of others’ emotions”, which evaluates the capacity to interpret emotions in others based on their verbal and visual cues; (3) “regulation of one’s own emotions”, which explores individuals’ capacity to regulate emotion through different activities, as well as their perceptions of control over their emotions; (4) “regulation of others’ emotions”, which explores one’s capacity to foster positive feelings in others; and (5) “utilization of emotion”, which assesses individuals’ ability to use positive emotions to help problem-solving (Davies et al., 2010). Each item is scored on a 5-point scale ranging from 1 (strongly disagree) to 5 (strongly agree). Higher total scores reflect higher levels of EI. The forward-backward translation approach was adopted to develop an Arabic version of the BEIS-10. The English version was translated to Arabic by a Lebanese translator who was completely unrelated to the study. Afterwards, a Lebanese psychologist with a full working proficiency in English, translated the Arabic version back to English. The translation team ensured that any literal and/or specific translation was balanced. The initial and translated English versions were compared to detect/eliminate any inconsistencies and guarantee the accuracy of the translation by a committee of experts composed of the research team, one psychologist, one psychiatrist and the two translators (Fenn et al., 2020). An adaptation of the measure to the Arab context was performed, and sought to determine any misunderstanding of the items wording as well as the ease of items interpretation; therefore, ensure the conceptual equivalence of the original and Arabic scales in both contexts (Ambuehl and Inauen, 2022). It’s important to note that the final version of the scale was developed in Modern Standard Arabic, rather than a country specific colloquial dialect, to ensure broad linguistic comprehensibility across Arabic-speaking countries. Care was taken to avoid Lebanese dialectical expressions and to use neutral, widely understood terminology commonly employed in formal written Arabic. After the translation and adaptation of the scale, a pilot study was done on 30 participants to ensure all questions were well understood; no changes were applied after the pilot study.

#### 2.4.3 Positive and negative affect schedule (PANAS)

Validated in Arabic (Narayanan et al., 2020), the Positive and Negative Affect Schedule (PANAS) (Watson et al., 1988) is used for measuring both positive and negative emotions. This instrument comprises 10 items to measure positive affect and 10 items to measure negative affect scored on a five-point Likert scale (*ω* = 0.91 for positive affect and 0.88 for negative affect). We chose PANAS as a measure to test validity based on past literature highlighting EI’s theoretical association with emotional experiences, especially positive affect.

### 2.5 Statistical analysis

#### 2.5.1 Confirmatory factor analysis (CFA)

We used data from the entire sample to perform a Confirmatory Factor Analysis (CFA) using RStudio, “lavaan” and “SemTools” programs (Jorgensen et al., 2022; Rosseel et al., 2023). We used Weighted Least Squares with Mean and Variance (WLSMV) estimation method, which is more appropriate for ordinal data (Li, 2016). Our intention was to test the original models of the BEIS-10 (i.e., five-factor model (Davies et al., 2010) and the one-factor model). The five-factor model is grounded in the theoretical framework of previous authors (Davies et al., 2010), who conceptualized EI as comprising five core components. We included the one-factor model as an alternative to test whether a general EI trait underlies the 10 items, as originally debated in the AES literature. Comparing both allows us to examine whether multidimensionality is justified or whether single latent construct best captures EI in this context. Multiple fit indices were calculated to assess model fit: the normed model chi-square (*χ*^2^/df; ≤5), the Root Mean Square Error of Approximation (RMSEA; ≤0.08), the Standardized Root Mean Square Residual (SRMR; ≤0.05), the Tucker-Lewis Index (TLI; ≥0.90) and the Comparative Fit Index (CFI; ≥0.90) (Byrne, 2013). Because WLSMV does not provide likelihood-based information criteria, supplementary analyses using the maximum likelihood ration (MLR) estimator were conducted solely to obtain the Akaike Information Criterion (AIC) and the Bayesian Information Criterion (BIC) values for model comparisons; if the difference between the two values is <2, both models are considered adequate, whereas if the difference between the values is >10, the model with the lower value is preferred (Vrieze, 2012). Multivariate normality was not verified as shown by the Mardia’s skewness and kurtosis values. Additionally, evidence of convergent validity was assessed using the Fornell-Larcker criterion, with Average Variance Extracted (AVE) values of ≥0.50 considered adequate (Malhotra et al., 2006). AVE was used to assess convergent validity only, consistent with its intended purpose. Discriminant validity was evaluated using Heterotrait-Monotrait ratio of correlations (HTMT) and inspection of latent factor correlations, as AVE alone in insufficient for assessing between-construct distinctiveness.

To examine gender invariance of the BEIS-10 scores, we conducted multi-group CFA (Chen, 2007) using the total sample. Measurement invariance was assessed at the configural, metric, and scalar levels (Vandenberg and Lance, 2000). We accepted ΔCFI ≤ 0.010 and ΔRMSEA ≤ 0.015 or ΔSRMR ≤ 0.010 as evidence of invariance (Chen, 2007; Cheung and Rensvold, 2002). Comparison of BEIS-10 scores between genders was done using the Mann–Whitney test.

Internal reliability was assessed using McDonald’s *ω* and Cronbach’s *α*; values greater than 0.70 reflecting adequate composite reliability (Malkewitz et al., 2023; Dunn et al., 2014). To assess concurrent validity, we examined bivariate correlations between the BEIS-10 scores and the PANAS subscales scores using the Spearman correlation coefficient. Based on Cohen (1992), values ≤0.10 were considered weak, ~0.30 were considered moderate, and ~0.50 were considered strong correlations.

## Results

CFA indicated that fit of the unidimensional model of the BEIS-10 was acceptable.

CFA indicated that fit of the 5-factor model of the BEIS-10 was acceptable: *χ*^2^/df = 124.60/25 = 4.98, RMSEA = 0.094 (90% CI 0.078, 0.111), SRMR = 0.034, CFI = 0.998, TLI = 0.996, AIC =. The standardized estimates of factor loadings of the one- and five-factor models were all adequate as shown in Table 2 respectively. However, inspection of the latent inter-factor correlations in the five-factor model (Table 3) revealed extremely high correlations between factors, several approaching unity, indicating substantial overlap and poor discriminant validity among the latent dimensions. These results suggest that the five-factor solution lacks empirical distinctiveness. Consequently, the unidimensional model was retained. The internal reliability of the total score was excellent (*ω* = 0.94, *α* = 0.94).

**Table 2:** Standardized estimates of factor loadings from the confirmatory factor analysis of the one- and five-factor models in the total sample.

Item number	One-factor model	Five-factor model
1. I know why my emotions change	0.77	0.79
2. I easily recognize my emotions as I experience them	0.84	0.85
3. I can tell how people are feeling by listening to the tone of their voice	0.84	0.86
4. By looking at their facial expressions, I recognize the emotions people are experiencing	0.86	0.88
5. I seek out activities that make me happy	0.84	0.85
6. I have control over my emotions	0.77	0.77
7. I arrange events others enjoy	0.76	0.74
8. I help other people feel better when they are down	0.83	0.80
9. When I am in a positive mood, I am able to come up with new ideas	0.87	0.87
10. I use good moods to help myself keep trying in the face of obstacles	0.84	0.83

Fit indices for the one-factor model: *χ*^2^ (35) = 162.83; SRMR = 0.039; RMSEA [90% CI] = 0.090 [0.077, 0.105], CFI = 0.997, TLI = 0.996, AIC = 11,575.88, BIC = 11,658.02; AVE = 0.68.

Fit indices for the five-factor model: *χ*^2^ (25) = 124.60; SRMR = 0.034; RMSEA [90% CI] = 0.094 [0.078, 0.111], CFI = 0.998, TLI = 0.996, AIC = 11,557.28, BIC = 11,680.49; AVE for factor 1 (= 0.68), factor 2 (= 0.76), factor 3 (= 0.66), factor 4 (= 0.60) and factor 5 (= 0.72). Appraisal of own emotions (items 1 and 2); appraisal of others’ emotions (items 3 and 4); regulation of own emotions (items 5 and 6); regulation of others’ emotions (items 7 and 8); utilization of emotions (items 9 and 10).

**Table 3:** Latent inter-factor correlation matrix of subscales of the five-factor model.

Variables names	1	2	3	4	5
1. Self-awareness	1	0.91	1.00	0.96	1.00
2. Self-regulation		1	0.94	1.00	1.00
3. Motivation			1	1.00	1.00
4. Empathy				1	1.00
5. Social skills					1

Values represent latent factor correlations estimated using CFA with the WLSMV estimator. Correlations approaching unity indicate poor discriminant validity.

### 3.1 Measurement invariance

All indices suggest measurement invariance at the scalar level (Table 4). No significant difference was found between males (median = 29; IQR = 15.75) and females (median = 30; IQR = 15) in terms of emotional intelligence, Mann–Whitney *U* = 19,274, *Z* = −1.32, *p* = 0.188.

**Table 4:** Measurement invariance of the Arabic version of the Brief Emotional Intelligence Scale across gender.

Model	CFI	RMSEA	SRMR	Model comparison	ΔCFI	ΔRMSEA	ΔSRMR
Model 1: One factor							
Configural	0.996	0.102	0.046				
Metric	0.996	0.100	0.048	Configural vs. metric	<0.001	0.002	0.002
Scalar	0.997	0.081	0.046	Metric vs. scalar	0.001	0.019	0.002
Strict	0.996	0.090	0.048	Scalar vs. strict	0.001	0.009	0.002
Model 2: Five factors							
Configural	0.997	0.103	0.039				
Metric	0.997	0.097	0.040	Configural vs. metric	<0.001	0.006	0.001
Scalar	0.998	0.078	0.040	Metric vs. scalar	0.001	0.019	<0.001
Strict	0.997	0.079	0.042	Scalar vs. strict	0.001	0.001	0.002

CFI, Comparative fit index; RMSEA, root mean square error of approximation; SRMR, Standardized root mean square residual.

### 3.2 Concurrent validity

Higher EI scores were significantly associated with higher positive (*rho* = 0.50; *p* < 0.001) and negative (*r* = 0.16; *p* < 0.001) affect (Table 5).

**Table 5:** Pearson correlation matrix.

Variable name	1	2
1. Emotional intelligence (BEIS scores)	1	
2. Positive affect	0.50***	1
3. Negative affect	0.16***	0.30***

***p < 0.001. Numbers in the table refer to Spearman correlation coefficients.

### 3.3 Discriminant validity

Discriminant validity was assessed using inspection of latent factor correlations and the Heterotrait-Monotrait ratio of correlations (HTMT), as recommended by recent guidelines. The five-factor BEIS-10 model showed extremely high latent correlations, ranging from 0.91 to values exceeding 1.00, indicating a lack of discriminant validity among the proposed dimensions. These results resulted in a non-positive definite latent covariance matrix, preventing reliable estimation of HTMT values. Such findings suggest that the five dimensions are not empirically distinguishable and reflect substantial conceptual overlap. Consequently, these results support the appropriateness of a unidimensional representation of emotional intelligence as assessed by the BEIS-10.

## Discussion

EI has been a widespread topic of scientific research and discussion among individuals and institutions over the last years, giving room for debate in regard to its measurement and applications (Prentice et al., 2020). The BEIS-10 is a brief, easy to administer, and cost-effective self-report measure that has seen growing use in assessing behavioral emotional intelligence (Davies et al., 2010), whose validity and reliability have been demonstrated in various samples and contexts [e.g., (Durosini et al., 2021; Hadadian-Chaghaei et al., 2021; Balakrishnan and Saklofske, 2015; Martín and Guzmán, 2012)]. The present study contributed to the literature by testing the fit of the originally proposed five-dimensions model of the BEIS-10, its internal consistency, construct validity and cross-gender invariance. The Arabic version of the scale showed good internal consistency and convergent and concurrent validity, along with an invariance across genders of the one-factor model that, in turn, produced the best fit to the data. These findings suggest that the Arabic BEIS-10 may be a valid and reliable self-administered measuring tool for the assessment of EI in Arab settings. Considering its simple and brief administration, this version of the scale might be of better convenience in Arab settings beset by shortage of resources and financial turmoil (Maalouf et al., 2019).

As for factorial validity, CFA provided evidence for a unidimensional solution, thus allowing a total score to be obtained that reflects the overall EI construct. This finding partly supports Davies et al.’s findings that their theoretically-driven five factors were found to be highly correlated suggesting a total EI score (Davies et al., 2010). In the present study, the extremely high inter-factor correlations observed in the five-factor model, several approaching unity, indicate a lack of discriminant validity and empirical distinctiveness among the proposed dimensions, thereby, providing strong statistical justification for the retention of a unidimensional model. Indeed, Davies et al. (2010) criticized their approach of including only two items in each of the five factors, as it is highly recommended by methodologists that, in a multidimensional scale, a minimum of three items load on each factor (Raubenheimer, 2004). Factors with fewer than three items are generally weak, unstable, and less likely to replicate (Little et al., 1999; Velicer and Fava, 1998). These considerations, along with cultural factors, may be among the reasons why there are discrepancies in factor structure between the present and the original validation study. A single first-order factor was found in Spanish students (Martín and Guzmán, 2012) and Southeast Nigerian adults (Amazue et al., 2015), concurring our findings. It is of note, however, that some previous studies were able to replicate the five-factor structure with good model fit in Canadian university students (Balakrishnan and Saklofske, 2015), Italian (Durosini et al., 2021) and Iranian (Hadadian-Chaghaei et al., 2021) community adults. It is noteworthy that the RMSEA value of both models were on the upper end of acceptable thresholds (≤0.08); RMSEA tends to be inflated in models with low degrees of freedom (df < 50) (Kenny and McCoach, 2003; Kenny et al., 2015). Since the df in our model was 25, model evaluation should rely on the other fit indices such as CFI and SRM (and not RMSEA), which have been shown to be more stable under these conditions (Lai and Green, 2016). In our study, both CFI and SRMR values were adequate, supporting a good fit despite the elevated RMSEA. The results indicated that both the five-factor and unidimensional models demonstrated acceptable fit indices. This finding suggests that the BEIS-10 can validly assess EI either as a multidimensional construct comprising distinct facets or as a general factor. The comparison of alternative models reinforces the robustness and flexibility of the BEIS-10 and adds to the literature by empirically supporting different structural interpretations of EI. The five-factor model showed a lower AIC value, suggesting marginally a better predictive fit, whereas the one-factor model demonstrated a lower BIC value, favoring a more parsimonious structure. Although the one- and five-factor models demonstrated acceptable fit, and given the negligible differences in comparative fit indices and the greater parsimony and interpretability of the one-factor solution, the unidimensional model was retained.

Excellent internal consistency of the BEIS-10 in its Arabic translation was evidenced by a McDonald’s *ω* of 0.94 and a Cronbach *α* of 0.94. Consistently, acceptable reliability coefficients for the total BEIS-10 scores were observed in students from Canada (*α* = 0.91) (Balakrishnan and Saklofske, 2015), USA (*α* = 0.83) (Howell and Miller-Graff, 2014), Spain (*α* = 0.69) (Martín and Guzmán, 2012), as well as in adults from Italy (*α* = 0.727) (Durosini et al., 2021) and Iran (*α* = 0.748) (Hadadian-Chaghaei et al., 2021). Beyond its good reliability, the Arabic BEIS-10 showed significant invariance across gender at the scalar, metric, and configural levels. The aforementioned, in line with the Arabic full-length version of the scale (Hallit et al., 2023), suggests that the scale’s structure is interpreted in a similar manner by males and females Arabic-speaking individuals. Establishment of measurement invariance across gender permits to make sure that differences between groups are not the result of distinct functioning of the BEIS-10 across groups, therefore enabling to perform future meaningful, sound and replicable in between-gender comparisons, and draw solid implications (Jeong and Lee, 2019; Nimon and Reio, 2011). Surprisingly, we could find no psychometric information on the BEIS-10 in terms of cross-gender invariance, thereby limiting its potential use in research addressing the question of gender differences in EI. We thus recommend that future studies test for measurement invariance across gender groups for the BEIS-10 before drawing any conclusions about gender comparisons using this measure.

Furthermore, greater scores on EI have been shown to be associated with higher affect, be it positive or negative, thus attesting to the concurrent validity of the Arabic BEIS-10. These findings are in agreement with previous psychometric studies on the full-length version, which also demonstrated a significant correlation between AES dimensions and positive and negative affect [e.g., (Satuf et al., 2020; Omar et al., 2014)]. These findings are in line with precedent literature on both ability and trait models of EI (Extremera and Rey, 2016; Kong et al., 2012; Zeidner et al., 2012b). However, it is important to note that higher negative affect among individuals with elevated EI does not necessarily reflect emotional dysregulation or poorer psychological functioning (Mohebbi et al., 2017). Rather, in the context of trait-based EI, this pattern may indicate heightened emotional awareness, sensitivity, and accuracy in identifying one’s affective states (Petrides et al., 2007). Individuals high in EI may experience and report emotions, both positive and negative, with greater clarity and intensity, resulting in elevated scores across affective dimensions (Gohm and Clore, 2002). This interpretation is consistent with theoretical models emphasizing emotional awareness and monitoring as central components of EI, particularly when assessed via self-report instruments (Petrides and Furnham, 2001), and suggests that EI may be associated with a broader and more nuanced emotional experience rather than the absence of negative affect (Mohebbi et al., 2017). As a matter of fact, the mental ability to maneuver affective experiences and information, that is EI, would participate in balancing emotions across these two poles (Pathak and Muralidharan, 2020). The emotional abilities that individuals with high EI retain are said to promote positive affect, aiding in the increase of one’s general cognitive evaluation of the satisfaction they have toward their own life (Kong et al., 2012).

Lastly, the unexpected positive correlation between EI and Negative Affect contrasts with theoretical expectations and prior research, which typically report a negative association between EI and NA (Mayer et al., 2008; Schutte et al., 2002). One possible explanation lies in the multidimensional nature of EI; individuals high in emotional awareness may report elevated negative affect due to heightened sensitivity to emotional states, without necessarily lacking emotion regulation skills (Petrides et al., 2007). Additionally, the BEIS-10’s brevity may limit its capacity to fully capture the regulatory components of EI, potentially skewing results toward perceptual aspects (Davies et al., 1998). Furthermore, individuals with high EI may be more adept at recognizing and managing their emotions, but this heightened awareness can sometimes lead to overthinking or rumination, especially in stressful situations. This can cause them to become more sensitive to negative emotional experiences, amplifying feelings of anxiety, sadness, or frustration (Davis and Nichols, 2016). Moreover, people with high EI may have an increased ability to manipulate emotions in ways that could be harmful or self-serving, contributing to negative outcomes in interpersonal relationships. In such cases, EI might be used strategically to manage emotions in a way that benefits the individual, but at the expense of others, fostering negative affect in social interactions or in the self (Davis and Nichols, 2016). Therefore, while EI can be a tool for emotional regulation, it can also be a double-edged sword, exacerbating negative emotions when used in maladaptive ways.

### 4.1 Practical implications

In terms of practical implications, the validation of the scale enables research on EI in diverse cultural contexts and enhances educational and workplace programs by tailoring emotional intelligence training to local needs. Furthermore, the BEIS-10 offers significant practical benefits across diverse settings. In Arab contexts, its application can be particularly valuable. In organizations, it can streamline recruitment by identifying candidates with strong emotional intelligence, crucial for leadership and teamwork. Educational institutions can utilize the BEIS-10 to assess students’ emotional competencies, informing curriculum development and personalized learning approaches. Furthermore, in therapeutic settings, the scale can provide valuable insights into clients’ emotional regulation, facilitating more effective interventions for individuals, couples, and families.

Lastly, the finding of gender invariance in emotional intelligence research challenges the assumption of inherent gender differences in this domain. This necessitates a shift in focus from broad gender-based generalizations to individual variations within each gender. Interventions and programs should be tailored to the unique needs and strengths of each individual, regardless of their gender. By acknowledging the potential for diverse EI profiles within both male and female populations, we can move beyond stereotypical assumptions and create more equitable and effective interventions that cater to the specific needs of all individuals. This approach can also help to dismantle harmful gender stereotypes and promote a more inclusive and nuanced understanding of EI across genders.

### 4.2 Limitations and research perspective

The current study presents certain limitations that must be addressed. The potentiality to extrapolate the findings and results has been restricted by the sole inclusion of a community sample of adults. Moreover, the sample was predominantly female (70.6%) and highly educated (78.8% with university degrees), which may have influenced participants’ responses on the BEIS-10 and limits the generalizability of the findings to the broader Arabic-speaking adult population. Subsequently, the appropriateness to administer the Arabic version of the BEIS-10 among clinical samples requires further research. In addition, the examination of some psychometric properties of the Arabic BEIS-10 (e.g., divergent validity and test–retest reliability) has not been achieved in the context of the present study, and thus calls for further investigation. Another limitation of the present study is the absence of a cross-cultural measurement invariance analysis. While the psychometric properties of the BEIS-10 were examined within a native Arabic-speaking population from Lebanon, this approach does not allow for the identification of potential cultural differences in how Emotional Intelligence is conceptualized and measured. Although the scale was translated into Modern Standard Arabic rather than Lebanese colloquial Arabic to ensure broad linguistic comprehensibility, subtle cultural and contextual differences across Arabic-speaking countries may still influence item interpretation. Given that Emotional Intelligence is culturally dependent, future research should aim to test the scale across diverse cultural contexts to establish broader cross-cultural validity and ensure that observed differences are not due to measurement bias. Future studies should also prioritize recruiting samples that are more balanced in terms of gender and education levels to better represent the general population and to examine whether the psychometric properties of the BEIS-10 hold across different demographic groups. Lastly, as cultural values have shown to navigate emotional expression, experience, and management (Pathak and Muralidharan, 2020), future studies are still needed to verify its psychometric properties and its cross-national invariance in samples from different Arab countries. This will also help recognize EI as culturally distinctive and aid in its exploration across cultural borders within the Arab world.

## Conclusion

The findings contended the said scale as adequate for the screening of EI in the Arab context. The utilization of the BEIS-10 as a short, easy-to-use and low-cost self-report measure ought to be beneficial for research and clinical purposes in Arab settings when it comes to assessing EI.

## Data Availability

The raw data supporting the conclusions of this article will be made available by the authors without undue reservation.
